# Barriers to Adoption of a Child-Abuse Clinical Decision Support System in Emergency Departments

**DOI:** 10.5811/westjem.18501

**Published:** 2024-10-14

**Authors:** Alanna C. Peterson, Donald M. Yealy, Emily Heineman, Rachel P. Berger

**Affiliations:** * University of Pittsburgh Medical Center, Pittsburgh, Pennsylvania; † University of Pittsburgh, Department of Emergency Medicine, Pittsburgh, Pennsylvania; ‡ University of Pittsburgh, UPMC Children’s Hospital of Pittsburgh, Division of Child Advocacy, Department of Pediatrics, Pittsburgh, Pennsylvania; § Pennsylvania Office of Children, Youth and Families, Harrisburg, Pennsylvania

## Abstract

**Introduction:**

Child abuse is a leading cause of morbidity and mortality in children. The rate of missed child abuse in general emergency departments (ED), where 85% of children are evaluated, is higher than in pediatric EDs. We sought to evaluate the impact of an electronic health record (EHR)-embedded child-abuse clinical decision support system (CA-CDSS) in the identification and evaluation of child maltreatment in a network of EDs three years after implementation.

**Methods:**

We anonymously surveyed all 196 ED attending physicians and advanced practice practitioners (APP) in the University of Pittsburgh Medical Center network. The survey evaluated practitioner awareness of, attitudes toward, and changes in clinical practice prompted by the CA-CDSS. We also assessed practitioner recognition and evaluation of sentinel injuries.

**Results:**

Of the 71 practitioners (36%) who responded to the survey, 75% felt the tool raised child abuse awareness, and 72% had a face-to-face discussion with the child’s nurse after receiving a CA-CDSS alert. Among APPs, 72% consulted with the attending physician after receiving an alert. Many practitioners were unaware of at least one function of the CA-CDSS; 38% did not know who completed the child abuse screen (CAS); 54% were unaware that they could view the results of the CAS in the EHR, and 69% did not recognize the clinical decision support dashboard icon. Slightly over 20% of respondents felt that the CA-CDSS limited autonomy; and 4.5% disagreed with the recommendations in the physical abuse order set, which reflects American Academy of Pediatrics (AAP) guidelines. Greater than 90% of respondents correctly identified an intraoral injury and torso bruise in an infant as sentinel injuries requiring an evaluation for abuse.

**Conclusion:**

A child-abuse clinical decision support system embedded in the electronic health record was associated with communication among practitioners and was overall perceived as improving child abuse awareness in our system. Practitioners correctly recognized injuries concerning for abuse. Barriers to improving identification and evaluation of abuse include gaps in knowledge about the CA-CDSS and the presence of practitioners who disagree with the AAP recommendations for physical abuse evaluation and/or felt that clinical decision support in general limited their clinical autonomy.

Population Health Research Capsule What do we already know about this issue?

*Early diagnosis of child abuse is critical to decrease morbidity and mortality. Abuse identification in general EDs may be assisted by clinical decision support.*

What was the research question?

*What are the benefits of and challenges to sustainability of a child abuse clinical decision support system (CA-CDSS) embedded in the electronic health record (EHR)?*

What was the major finding of the study?

*Three-quarters of practitioners reported the CA-CDSS increased child abuse awareness and prompted interdisciplinary interactions.*

How does this improve population health?

*Using an EHR-embedded CA-CDSS may be one approach to improving child abuse awareness in general EDs, thereby decreasing abuse-related morbidity and mortality.*

## INTRODUCTION

Child maltreatment causes significant morbidity and mortality in the United States, especially for children under four years of age. Over three million children each year are reported to child protective services (CPS), and 1,600 children die at the hands of caregivers due to maltreatment,^1^ a number greater than children who died from COVID-19 during the first 2½ years of the pandemic.^2^ The number of children dying from maltreatment has been increasing; there was a 10% increase in fatalities from 2016–2021.^1^ ^,^ ^3^ Between 20–25% of children who are ultimately diagnosed with physical child abuse have been previously seen by a medical practitioner who failed to identify the abuse.^4^ ^–^ ^7^ Appropriate recognition and evaluation of physical child abuse in general emergency departments (ED), where most children receive emergency care, is crucial.

To improve the quality of identification, evaluation, and reporting of child maltreatment in the University of Pittsburgh Medical Center (UPMC) general EDs, we developed and deployed a child-abuse clinical decision support system (CA-CDSS) in our electronic health record (EHR) starting in 2016.^8^ ^–^ ^10^ At that time there were 13 general EDs with the hospital management software Cerner (Cerner Corp, Kansas City, MO) in the UPMC system; they went live with the CA-CDSS system between January–March 2016. As the hospital system acquired additional EDs (one each in June 2017 and April 2019, and four in September 2019), they were added to the CA-CDSS.

Prior to the go-live at each site, training occurred for both ED nurses and practitioners. Nurses completed an interactive online learning module, which remains part of the onboarding process for ED nurses and has become an education requirement every two years. Practitioner education was done through the ED medical directors. Prior to the go-live at each hospital, each ED medical director received an onboarding packet that included general child abuse education, screen shots of all parts of the CA-CDSS, case examples, and a way for practitioners to reach out with questions. Each ED medical director also met with one of the authors (RB) who reviewed the onboarding packet and answered questions. The medical directors were, and continue to be, responsible for disseminating education to their practitioners. In addition to the initial training, ongoing training includes feedback to practitioners about cases from the medical director of each ED site-specific trainings at standing practitioner meetings in individual EDs and bi-monthly systemwide conference calls, which use case examples as a springboard for discussing specific child abuse-related topics. These calls provide continuing medical education credit for practitioners.

The features of this tool include a set of triggers including a child abuse screen (CAS) completed by the primary nurse, an alert that practitioners receive when a patient has triggered the CA-CDSS, and a physical abuse order set to assist practitioners in ordering the correct testing based on patient age and injury.^8^ ^,^ ^9^ In addition, triggering the CA-CDSS results in an icon appearing on the main ED dashboard next to the patient’s name. When providing feedback to practitioners about cases in which the physical abuse order set wasn’t used when it was indicated, one of the co-authors (AP), who is also the director of one of the general EDs, noted that some practitioners reported that they did not agree with the recommendations in the order set and preferred to use clinical judgment.

We sought to understand the barriers to compliance with the order set recommendations, assess the impact of the CA-CDSS, and identify opportunities to improve the CA-CDSS with the goal of increasing engagement with the CA-CDSS overall.

## METHODS

### Setting

The 19 general EDs in the UPMC hospital system operate in urban and rural settings and include community hospitals and academic centers. The primary academic centers are in the city of Pittsburgh, PA. The remainder are EDs affiliated with community hospitals across much of Pennsylvania and with individual sites in New York. Annual practitioner turnover at these 19 general EDs for full-time employees averages 6.8% for APPs and 3.6% for physicians. There is a total of ∼30,000 ED visits for children <13 years of age (the age included in the CA-CDSS) at the 19 EDs annually; the proportion of all ED visits involving children ranges from 1–3% at the academic sites and up to 12% in the community sites.

### Survey

In February 2020, our team (a general emergency physician who is the director of one of the hospital system’s EDs [AP] and a child abuse pediatrician [RB] from the affiliated children’s hospital that was not one of the 19 included hospitals) emailed a survey to all 196 attending physicians and advanced practice practitioners (APP) at the 19 UPMC EDs. The email provided an anonymous link to a 25-question, web-based survey (Qualtrics LLC, Seattle, WA) that used skip logic, meaning clinicians received only questions that were relevant based on previous responses (Appendix A). Self-reported demographic data included years in practice, hospital affiliation(s), and practitioner type (physician or APP). The survey aimed to assess the practitioner’s 1) knowledge about the CA-CDSS and its associated functionality; 2) engagement with and attitudes toward the CA-CDSS; 3) recognition of sentinel injuries—minor injuries that necessitate an evaluation for physical abuse; and 4) reasons for not using the physical abuse order set even when it was indicated. The survey was designed so that practitioners would learn about the CA-CDSS as they completed the questions.

### Statistical Analysis/Measures

We used descriptive analyses to measure the proportion of surveys completed, knowledge of practitioners about the CA-CDSS, attitudes toward the CA-CDSS, recognition of injuries that should raise concern for physical abuse, and barriers to evaluating and reporting suspected abuse.

### Ethical Consideration/Approval

The UPMC Quality Improvement Committee approved this project. There was no formal ethics review, and no potential conflicts of interest were identified.

## RESULTS

### Response Rate and Practitioner Characteristics

There was a 43% (84/196) initial response rate, with 13 surveys excluded for lack of completeness, leaving 71 surveys (36%) for analysis. Of the 13 incomplete surveys, one practitioner wasn’t eligible and 10 of the remaining 12 incomplete surveys had fewer than 35% of the questions answered. As a result, we chose to exclude them entirely. Most respondents were physicians who worked in community EDs and had more than 15 years of experience (Table 1).

**Table 1.** Demographic characteristics of survey respondents.

Demographic characteristics of survey respondents	# (%) of respondents with completed surveys (N = 71)	Characteristics of surveyed population (n = 196)
Practitioner type		
Attending physician	57 (80%)	147 (75%)
APP	14 (20%)	49 (25%)
Primary practice type^*^		
Academic	26 (37%)	74 (38%)
Community	38 (54%)	122 (62%)
Split between academic community	5 (7%)	
Years of experience		
>15 years in practice	30 (42.3%)	Not available^^^
6–15 years in practice	27 (38.0%)	Not available
0–5 years in practice	14 (19.7%)	Not available

* Two declined to state.

^ Years of experience was collected as part of the survey and, therefore, was not available for practitioners who did not complete the survey.

*APP*, advanced practice practitioner.

### Practitioner Knowledge About the CA-CDSS and Its Associated Functionality

#### The child abuse screen (CAS)

Of the 71 respondents, 27 (38%) did not know who completes the CAS, and 54% (38/71) were unaware that they could see the completed CAS (vs simply being alerted when it was positive).

#### The alert

The same proportion of practitioners (27/71) did not recall ever seeing an alert, and 69% (49/71) of practitioners did not know that the lightbulb icon on the ED dashboard meant that the patient had triggered the CA-CDSS.

### Practitioner Engagement with and Attitudes Toward the CA-CDSS

#### The alert

Of the practitioners who remembered seeing the alert, 68% (30/44) reported that they always approached the child’s nurse for further details, and 86% (12/14) of the remaining practitioners reported that they sometimes approached the nurse; two could not recall whether they had done so. Of the 11 practitioners who were APPs and recalled seeing the alert, 78% (8/11) reported that they always approached the attending physician to discuss the case, and the other three reported they sometimes did.

#### The emergency department child physical abuse order set

Forty-two percent (30/71) of respondents reported having used the physical abuse order set. Of the 58% who did not report using it, 34% (14/41) indicated they were unaware of it, 54% (22/41) believed the order set was not relevant for the patient(s) they were treating, and 5% (2/41) were unable to find it. One practitioner made a broad comment about not using any order sets because he wanted to “use my brain” and not follow recommendations. Half (33/66) of respondents indicated they agreed with the recommendations contained in the order set, 45% (30/66) were neutral, and 4.5% (3/66) disagreed with the recommendations.

#### Attitudes

**Figure 1.**
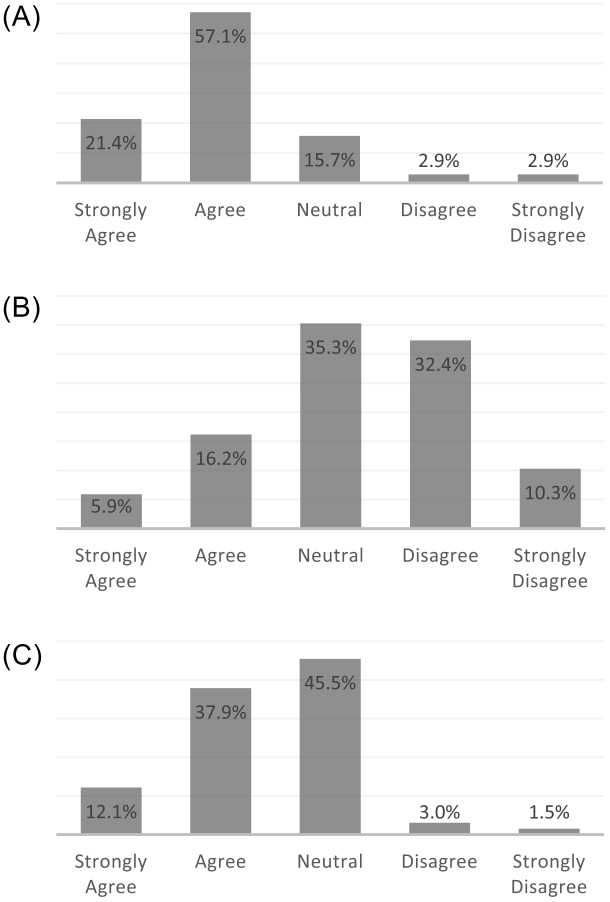
Practitioner attitudes about the alert’s effect on clinical decision-making: a) Practitioner responses to the statement, “The CA-CDSS increases my awareness of the potential risk for child abuse,” (n = 70); b) Practitioner responses to the statement, “The CA-CDSS pop-up alert and ED physical abuse order set limit my ability to make independent decisions,” (n = 68) and c) Practitioner responses to the statement, “I agree with the suggested evaluations/workup in the ED physical abuse order set.” (n = 66). *CA-CDSS*, child-abuse clinical decision support system; *ED*, emergency department.

**Figure 2.**
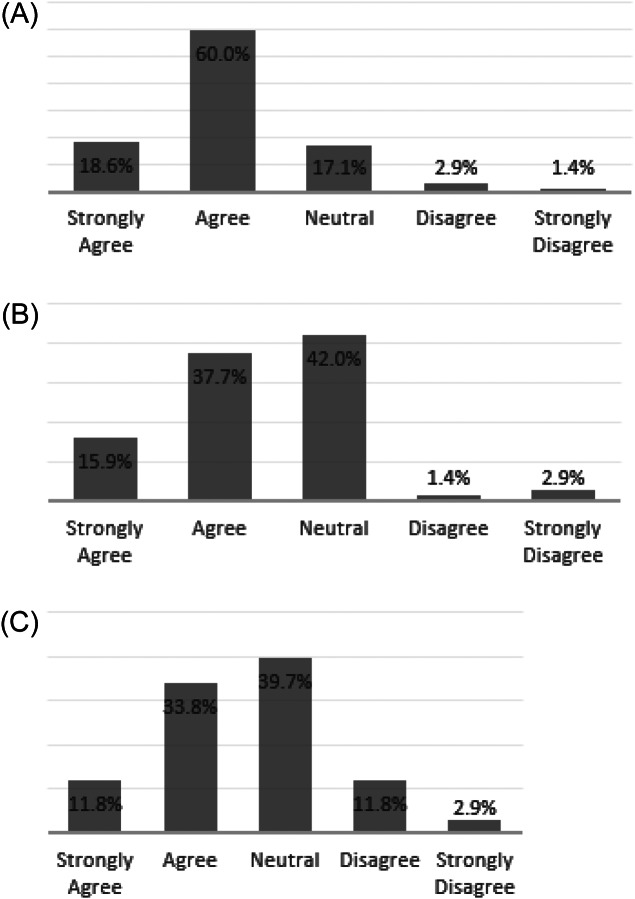
Practitioner attitudes about the alert and order set: a) Likert scale practitioner responses to the statement “The pop-up alert I receive from the CA-CDSS is clearly worded,” reported as a percentage of 70 respondents (1 declined to state); b) Likert scale practitioner responses to the statement “Using the ED physical abuse order set fits well in my clinical workflow,” Reported as a percentage of 69 respondents (two declined to state); and c) Likert scale practitioner responses to the statement “The ED physical abuse order set saves time when evaluating patients,” reported as a percentage of 68 respondents. (Three declined to state.) *CA-CDSS*, Child-abuse clinical decision support system; *ED*, emergency department.

Overall attitudes about the CA-CDSS were positive, with 79% of practitioners agreeing or strongly agreeing with the statement, “The CA-CDSS increases my awareness of the potential risk for child abuse” (Figure 1a). Twenty-two percent of respondents felt that the CA-CDSS alert and physical abuse order set limited their ability to make independent decisions (Figure 1b). More than 75% of respondents felt that the alert was clear (Figure 2a), that the alert and order set fit well into practitioner workflow (Figure 2b), and that it saved time (Figure 2c).

#### Recognition of injuries that do and do not necessitate a physical abuse evaluation

Case 1 described a 13-day-old with a subconjunctival hemorrhage clearly documented at birth. This case was an example of an infant who did not need a child abuse evaluation. Almost 40% of respondents (26/71) incorrectly stated that this required a child abuse evaluation.

Cases 2 and 3 described a 4-month-old and 2-month-old with an intraoral laceration and torso bruise, respectively. These cases were used to demonstrate infants who require a child abuse evaluation. In these scenarios, 91% (56/61) of practitioners in case 2 and 97% (69/71) in case 3 correctly noted the need for a child abuse evaluation.

#### Barriers to evaluating and reporting suspected physical abuse

**Figure 3.**
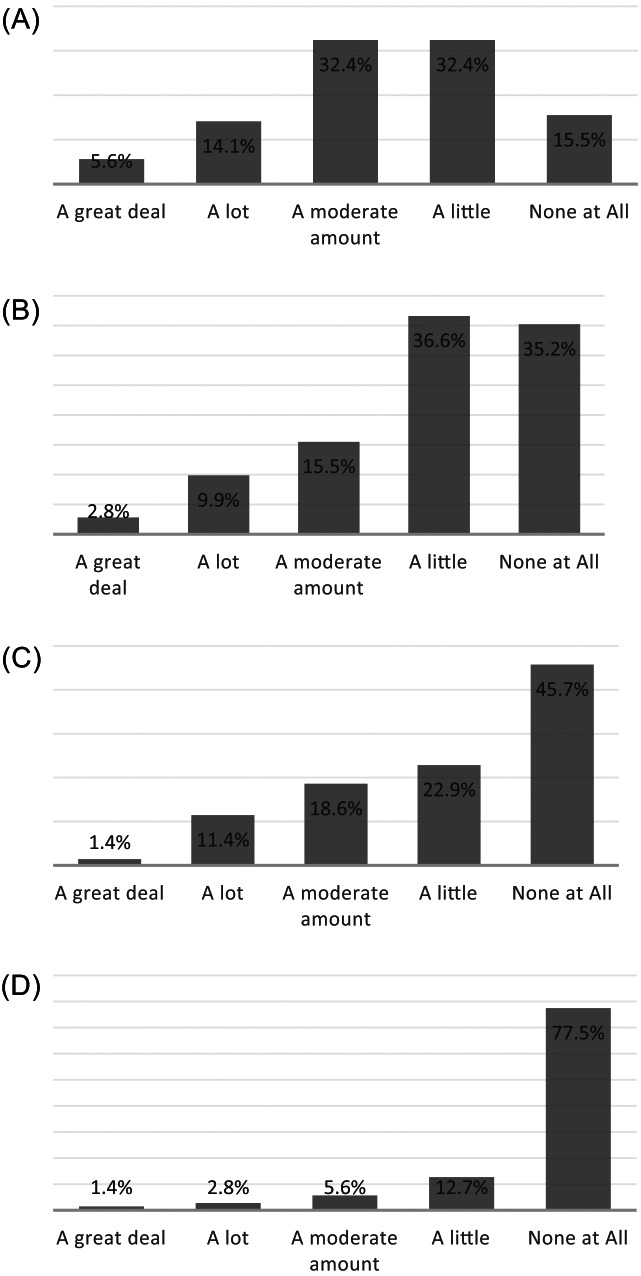
Practitioner self-reporting about barriers in child abuse recognition and evaluation: a) Lack of certainty about when to be concerned for abuse; b) Lack of certainty about what tests are indicated; c) Lack of social worker or ancillary support; and d) concern about being called to court.

Of respondents, 89% (60/71) expressed uncertainty regarding their ability to recognize child abuse, with 52% reporting at least moderate amount of uncertainty in recognition (Figure 3a). When asked about pursuing the appropriate evaluation for child abuse, 65% (46/71) expressed uncertainty as to which tests were indicated (Figure 3b). Lack of a social worker or ancillary support in the ED was identified as a barrier by 54% (38/70) of respondents. Too much time needed for the workup was identified as a barrier for 16% (11/71) of respondents. Lastly, concern about being called to court to testify was identified as a barrier by 10% (7/71) of respondents.

#### Free-text responses regarding the child abuse-clinical decision support system

The final question allowed for respondents to provide free-text comments and/or suggestions about the CA-CDSS. A total of 18 (25%) respondents entered a free-text response. A common response related to the subtleties in these types of cases and the desire to have the ability to consult a pediatric and/or child abuse specialist while the patient is in the ED. As discussed below, practitioners do have this access but were unaware of it.

Sometimes I have trouble choosing an order set because the situation does not fit in the list provided….

[Practitoner] uncertainty around the specifics/subtleties related to a specific case can be a big factor in choosing to fully utilize the order set and/or to file a report. I think [practitioners]/ patients/ families would greatly benefit by more direct involvement with the child advocacy team [while] the patient is in the emergency department.

Raise awareness to [practitioners] so that they can consult pediatric specialists including peds [emergency] physicians for further assistance with suspected child abuse cases.

A second theme was not related specifically to the CA-CDSS but to the more general issues of pop-up fatigue” (ie, alert fatigue):

More pop-ups cause click fatigue. This has destroyed medicine.

We suffer from pop-up fatigue and this can just be one more.

A third set of comments were related to the desire to learn more about the CA-CDSS itself:

I was not aware of this tool, so please send out an email describing it.

Make clinicians aware of this tool.

…more webinars or collaboration with…experts would be great.

If there are routine free recorded lectures for us on classic regional cases….I would be interested in taking them…

## DISCUSSION

This is the first paper to evaluate the long-term effect of an EHR-embedded CA-CDSS on patient care in a network of general EDs. The most encouraging observation is that the CA-CDSS appears to facilitate interdisciplinary communication between nurses, physicians, and APPs. One of the concerns about transitioning the CAS from a paper form—the way in which it was originally studied and validated^11^—to an electronic format was that multidisciplinary communication would no longer be required and, therefore, would not occur.

The fact that 91% and 97% of practitioners, respectively, correctly identified an intraoral injury and a bruise as sentinel injuries and recognized the need for a child abuse evaluation is encouraging given the literature suggesting that practitioners often do not recognize these more subtle forms of abuse.^12^ ^–^ ^14^ It is not possible to know whether this knowledge is related to the presence of the CA-CDSS or the practitioner education that has supplemented the CA-CDSS.

Over 90% of practitioners stated that concern about being called to court had little or no impact on their decision to report/evaluate for abuse. This is encouraging; in a landmark study by Flaherty and colleagues,^15^ “Clinician spent many hours in court testifying” was one of the practitioner characteristics identified in a significant proportion of the practitioners who did not report to CPS despite having concern for maltreatment. Each of the authors of the current manuscript has personally spoken with practitioners who have said that they do not want to get involved in the legal system. Our data suggests that, overall, this concern is not driving decision-making about whether or not to make a report to CPS in our general EDs.

Our results also underscore the need for usability of the CA-CDSS; practitioners felt the alert and orders suggested were clear and useful, fit well into workflow, and saved time. The importance of usability cannot be overemphasized: if this is poor then it is unlikely that practitioners will use the tool.^16^ However, as other responses to our survey seem to demonstrate, usability is necessary, but not sufficient, for practitioner engagement with the CA-CDSS. The number of practitioners unaware of different aspects of the CA-CDSS including the icon, the alert, and the order set demonstrates the challenges in education about a low-frequency event for any given practitioner in a large hospital system, even with a relatively low practitioner-turnover rate.

Improving the knowledge of practitioners about the CA-CDSS itself is likely to be challenging. There are multiple studies evaluating the effectiveness of different training modalities, including hands-on training in a laboratory setting, required training, use of “super users” to assist others in learning, and use of “just-in-time” training.^17^ ^–^ ^20^ But all these approaches are challenging when they aim to address a relatively rare event. While child abuse is a common cause of morbidity and mortality overall, it is not commonly seen or recognized by most individual practitioners. Multiple training approaches as well as individualized follow-up and ongoing training have all been shown to sustain engagement; these are approaches we have used and continue to use.^17^

Despite these approaches, many of the free-text responses noted an unawareness of the existing resources (eg, access to pediatric experts and ongoing available education). We hypothesize that some of this lack of awareness may be related to the increase in the use of locum tenens and temporarily or casually employed practitioners, which was rare prior to COVID-19 but increased significantly during the pandemic and has continued. These practitioners rarely if ever attend the education sessions and do not receive onboarding like other practitioners, both of which can limit the real utility of resources positioned to aid with knowledge and care.

It is concerning that one of the most common free-text responses related to the desire to have real-time access to a pediatric and/or child abuse specialist while the patient is in the ED. Emergency clinicians in all the general EDs in the hospital system already have this access. There is a phone number they can call 24/7, which provides consultation with a pediatrician at the tertiary-care pediatric hospital in the hospital system. If the pediatric practitioner is unable to answer a child abuse-specific question, the child abuse specialist on call is paged. The lack of knowledge about this phone number reflects a more general lack of knowledge about hospital resources. As a result of this survey, an email was widely distributed to make practitioners aware of this phone number, and it is now included on flow sheets in multiple EDs.

Many of the free-text responses targeted CDS tools in general rather than specifically the CA-CDSS. Alert fatigue was mentioned in the free-text responses regarding the CDS tool and are consistent with prior studies, which demonstrated that interruptive alerts (eg, pop-ups) adversely affect practitioner use of recommendations from CDS.^21^ ^–^ ^24^ Both free-text comments related to the alert (“More pop-ups cause click fatigue. This has destroyed medicine,” and “We suffer from pop-up fatigue and this can just be one more”) are consistent with the broader issue of alert fatigue. While the low trigger rate for the CA-CDSS is highly unlikely to be a major contributing factor to alert fatigue, it may exacerbate pre-existing frustration with the alerts/pop-ups in general.

Reported successful interventions to combat alert fatigue include the persistent presence of the pop-up on the chart until it is acknowledged, as opposed to having multiple alerts for the same clinical concern.^24^ While a persistent alert wasn’t a possibility in our EHR at the time the CA-CDSS was developed, we designed the system so that each practitioner only receives one alert in response to the specific concern of trying to alleviate alert fatigue. Interestingly, some of the early feedback about the system was that some practitioners wanted to be alerted repeatedly until they decided whether to evaluate for abuse, at which point they wanted to be able to silence the alert. It is not possible for the current EHR system to customize alerting rules for different practitioners; when designing any given CDS system, a decision must be made about the timing and frequency of alerts that applies to all practitioners. Addressing the issue of alert fatigue is challenging because it is the sum of all alerts rather than the alert from any single system that impacts adoption rates for all CDS. As a result, developing a solution requires a hospital/hospital system to holistically evaluate all the CDS systems in use.

Multiple conversations with practitioners displayed little agreement about when in the workflow and how often the alerts should be provided. Some practitioners wanted to know at first chart open that they should be concerned about abuse, so that they have this in mind when they examine the child and speak with the family, while other practitioners want the information later in the visit when they are formulating a differential diagnosis. Some practitioners wanted to be alerted only once, while others preferred to be alerted repeatedly until they decided whether to evaluate for abuse.

The finding that over 20% of practitioners perceive CDS as a threat to physician autonomy likely has a significant impact on engagement and acceptance of any CDS. The concern about CDS impacting practitioner autonomy is not specific to child abuse CDS; rather it is a major barrier to CDS in general.^21^ ^,^ ^25^ ^–^ ^27^ Interestingly, and perhaps surprisingly, the characteristics of the 15 clinicians who felt that CDS was a threat to physician autonomy were not different from the clinicians who did not have this sentiment in terms of their response to the questions about how often lack of certainty about when to be concerned about abuse or lack of certainty about what tests to do influenced their decision about whether to do a physical child abuse workup. Neither did they differ from other practitioners in terms of the proportion who correctly answered the three scenarios related to sentinel injuries. While it is difficult to interpret this data given the small numbers, it suggests that there is a need to better understand how these practitioners would prefer to receive assistance making high-quality, evidence-based decisions since they recognize that they are uncertain and that this impacts their clinical decision-making, but they don’t see CDS as a solution.

Behavioral economics (BE), an evolving field rooted in economic and psychology, may be one approach to enhance physician engagement with the CA-CDSS. Behavioral economics is based on recognition that humans are not rational decision makers and rarely behave as the conventional economics theory would predict. Interventions informed by BE attempt to change physician practice using a “nudge,” an intervention that predictably changes human behavior without significantly limiting free choice or changing financial incentives. Changing default settings and providing social reference points (eg, peer comparison) are most consistently effective interventions in improving physician practice as it relates to following evidence-based practice.^28^ ^–^ ^30^ As it relates to a CA-CDSS, BE may be able to be used to nudge practitioners to follow AAP recommendations for physical abuse, for example, by providing feedback about their performance compared to their peers.^28^ ^–^ ^30^ Importantly, BE focuses on the subset of practitioners who are amenable to “nudging,”^31^ which is generally only a subset of practitioners. One would not expect a practitioner who has a negative view of CDS (ie, feels that it limits autonomy and does not improve patient care) to be amenable to nudging, but it may still offer some ability to improve engagement.

Perhaps the most concerning and confusing finding was how many practitioners either have a neutral or negative opinion of the evidence-based AAP recommendations for child physical abuse evaluation. This observation occurred alongside 65% expressing uncertainty about how to evaluate for physical abuse and more than 90% recognizing that sentinel injuries require a physical abuse evaluation. Requiring a user to select a reason for not following recommendations provided within the alert (eg, to follow the recommendations in the physical abuse order set) can increase compliance with recommendations^32^ and may provide insight into these seemingly inconsistent responses. Implementation of a required response needs to be weighed against the possibility of generating negative attitudes toward the tool and perceived impairment of workflow. Another way to potentially increase adherence to AAP recommendations is to ensure that practitioners understand how the CA-CDSS functions and ensure that practitioners understand the source for the electronic tool recommendation^27^; we currently do this during bi-monthly education sessions with practitioners.

## LIMITATIONS

There are several limitations to the study. The response rate was relatively low, and those who chose to respond may not be representative of all practitioners. The only demographic data available for all practitioners was whether they were a physician or APP and the type of hospital they practiced in; for these characteristics, respondents and non-respondents looked similar. We felt it was important for the survey to be anonymous so that respondents would be comfortable providing honest feedback; this approach means we could not target non-respondents to improve the response rate. It is also possible that the respondents having particularly strong feelings—positive or negative—about the CA-CDSS were most likely to respond to the survey. Because of the low number of practitioners who responded to the survey from any single hospital and because practitioners can work at more than one hospital within the 19-hospital network, it was not possible to determine whether there were site-specific variations in the opinions of practitioners about the CA-CDSS. Finally, the responses to the survey may not reflect actual practice and instead may reflect what practitioners know they should do. For example, practitioners may say they speak with the nurse when a child has a positive CAS, but it is not possible to know if this actually occurs.

## CONCLUSION

In summary, our data suggests that a child-abuse clinical decision support system embedded in the electronic health record has yielded positive results in both interdisciplinary communication and practitioner attitudes toward the tool, including perceiving the tool as increasing child abuse awareness. However, there remain gaps in knowledge of the CA-CDSS functionality and in compliance with the recommendations. Comments suggest that practitioner dislike of CDS tools in general, and specifically alerts delivered in pop-up form, may contribute to poor adherence. While limitations of the EHR limit the type of alert, CA-CDSS educational efforts could be augmented to specifically address perceived barriers to autonomy and possibly to include behavioral economics techniques, such as peer comparison or testimonials,^33^ ^–^ ^35^ to improve compliance with American Academy of Pediatrics recommendations. Further research should focus on the effectiveness of these interventions as we continue to improve care for a rare event that carries significant morbidity and mortality.

## Supplementary Information




