# Supplementary material for: Barriers to Adoption of a Child-Abuse Clinical Decision Support System in Emergency Departments
Source: West J Emerg Med. 2024 Oct 14;25(6):1011–9. doi: 10.5811/westjem.18501 (PMC11610743; doi:10.5811/westjem.18501)
Supplement: Supplementary file 1 [file wjem-25-1011-s001.docx]

Appendix A: Survey sent to ED providers

We would like your feedback about the UPMC Child Abuse Clinical Decision Support system so we can improve it. This will take you less than 10 minutes to complete. Thank you.

1. What is your current role in the ED?

Attending CRNP/PA Other, please specify _____________________________

1. Which ED(s) do you currently work at?

(all EDs listed with providers able to select multiple)

1. How long have you been in practice, post-training?

0-5 years 6-15 years >15 years

1. Do you know that the primary nurse completes the Child Abuse Screen for all children under age 13? (screen shot of CAS included)
   Yes No, but now I do!
2. Do you know that you can see the results of the Child Abuse Screen (CAS) in GenView? (screenshot of GenView with results of a CAS)

Yes No, but now I do!

1. Do you remember ever receiving the pop-up alert below on a patient? (screenshot of pop-up alert)

Yes No

1. When you see the pop-up alert on a patient, do you speak to the child's nurse?

Yes, I always do Yes, I sometimes do No Don't remember

1. Do you discuss the pop-up alert with your attending physician?

Yes, I always do Yes, I sometimes do No Don't remember

1. Did you know that the lightbulb icon on the tracking board means that the patient has alerted the child abuse alert system either because the CAS is positive or because other documentation (e.g. chief complaint) suggests that the child is at risk for abuse? (screenshot of lightbulb included)

Yes No, but now I do!

1. Have you ever used the ED Physical Abuse PowerPlan?

Yes No

1. If you have not used the ED Physical Abuse PowerPlan, why not? (check all that apply)

Not relevant for patient

Did not agree with the recommendations

Was unable to find it

Did not think it would be useful

I could not figure out how to use it

Did not know we had a PowerPlan

Other, please explain____________________________

1. Please use the scale below to rate your agreement with the following statements about the child abuse clinical decision support system (CA-CDSS).

Strongly Disagree Disagree Neutral Agree Strongly Agree

The CA-CDSS increases my awareness of the potential risk for child abuse.

The pop-up alert I receive from the CA-CDSS is clearly worded.

The pop-up alert and ED Physical Abuse PowerPlan are useful tools at the point of care (i.e. it is appropriate/improves quality of patient care).

Using the ED Physical Abuse PowerPlan fits well in my clinical workflow.

The CA-CDSS pop-up alert and ED Physical Abuse PowerPlan limit my ability to make independent decisions.

The ED Physical Abuse PowerPlan saves time when evaluating patients.

I agree with the suggested evaluations/workup in the ED Physical Abuse PowerPlan.

Sentinel injuries are injuries which are highly associated with physical abuse and with escalating violence in children <6 months of age. These injuries warrant an evaluation for abuse whenever they are seen in this age-group except in the rare circumstance that they occurred in a public place/were witnessed by unrelated adults. Which of the following would you consider to be a sentinel injury that requires a physical abuse work-up?

1. An 11-day old girl presents with subconjunctival hemorrhage. Parents report that it was there at birth, which you confirmed through review of the birth records.

This is a sentinel injury. Yes No

This requires an evaluation. Yes No

1. A 4-month old boy presents with a frenulum tear. Parent reports that he cut his mouth on the clip of a pacifier.

This is a sentinel injury. Yes No

This requires an evaluation. Yes No

1. A 2-month old boy presents with a bruise on his torso. Parents don't know how he got it but say he seems to bruise easily.

This is a sentinel injury. Yes No

This requires an evaluation. Yes No

1. How much do the following factors influence your decision whether to do a physical child abuse work-up? (None at all, A little, A moderate amount, A lot, A great deal)

Lack of certainty about when to be concerned for abuse

Lack of social worker or ancillary support

Lack of certainty about what tests are indicated

Too much time needed for the work-up

Concern about being called to court

1. Do you have any suggestions for training or ideas for improving the CA-CDSS?
2. Please provide any other comments you would like to share with us about the CA-CDSS.
